# The Judicial Treatment of Infanticide

**Published:** 1898-06-11

**Authors:** 


### Modern Sociology.

### THE JUDICIAL TREATMENT OF INFANTICIDE.

The right method of dealing judicially with the crime of infanticide is undoubtedly one of the most difficult problems with which our legal authorities have to cope, and the question has lately arisen in many quarters as to whether it has hitherto been successfully met. In the opinion of those who have had opportunities of forming a sound judgment on the subject, there has been an error up to the present in the direction of too great leniency in the case of women—often quite young girls, who have put their infants to death—in the majority of instances a verdict of "concealment of birth" having been returned instead of "wilful murder," although the one offence is most frequently the precursor of the other. Generally, also, the violent death of the child is not designated as murder, unless a very considerable period of time has elapsed from the date of its birth. Now, it must be remembered in this connection, that the punishment of criminals and the safety of society are not by any means the only functions pertaining to the administration of justice; its highest prerogative may, indeed, be rightly held to consist in the education of the lowest classes—by the action of the law—on the distinction between right and wrong. At this time, it is a simple fact that they see no guilt whatever in flagrant misdeeds which the laws of God and man alike condemn with unquestionable severity—and of crimes which the so-called dangerous classes hold to be perfectly reasonable and justifiable, infanticide is perhaps the most notable example. For this reason undue leniency in the action of our law courts towards a woman who kills her own child is much to be regretted. It is a most natural tendency on the part of judges and magistrates, and one which most persons would be likely to share who do not understand the subject in all its bearings. There are, indeed, great grounds for compassion in the case of some unfortunate girl, deceived by the man she loved and trusted, finding herself burdened with an infant whose existence renders it impossible for her to go to service or earn her own living respectably. Yet that she should rid herself of the incumbrance by the murder of the hapless being to whom she has given birth, is to outrage the strongest and most sacred instinct which God has implanted in the heart of woman—the instinct of motherhood. It must not be forgotten that, in all such cases, there is a refuge both for the unwedded mother and the illegitimate babe in the workhouse of her parish. In nine cases out of ten it is an unwillingness to enter the Union which makes the fallen girl prefer the death of her child by her own hands to a course which is so distasteful to herself. Those who have carefully studied this painful question feel strongly that there ought to be no limitation as to the time, longer or shorter, which has followed the birth, in classifying the destruction of a child by its mother as a definite crime of murder. Most of these cruel acts take place on the day of the infant's birth, and, as a rule, the woman is in full possession of her senses. Of course, where it can be shown that there has been delirium as a result of the confinement the case is taken out of the category of crime altogether, but such instances are comparatively rare among the lower orders. We must all of us have been continually shocked by hearing of healthy young women, whose robust physical strength enabled them to walk about as usual within a few hours of their delivery, who were found to have concealed their murdered infant in their box, and had carried it out, and thrown it into the nearest pond. There is less excuse for such criminal acts in these benevolent days than even the dread of the workhouse can afford, because there are now many societies which have been founded with the view of saving these unhappy young mothers from deeper sin and degradation by undertaking the maintenance of their infants, if they will enter a Home until they can be placed in service, and so become able to earn money for the child's support. Other consequences of their fall which are supposed to drive erring girls to the destruction of their offspring, such as the shame and disgrace of their position, the anger of respectable relations, the estrangement of friends, are the inevitable penalties which the Divine Justice has decreed should follow sin; and the human authorities who wield judicial power, do ill in helping the fallen women to evade them by retarding the recognition and punishment of the crime, and indeed sometimes by ignoring it altogether. Were there a somewhat more active and stringent investigation into the cases technically denominated "concealment of birth," it would be found that the number of infants yearly sacrificed in this manner within the borders of our own country is simply enormous, and far beyond what our legislators could for a moment believe to be really the case. This holocaust will continue to be offered to the selfishness and moral degradation of fallen women, so long as it is the almost invariable rule in our law courts that the destruction of a child on the day of its birth shall not be considered the result of an act of murder. A very light sentence of a few days' imprisonment, and often not even this, is passed on the girl, and there the matter ends, with the conviction left on her mind that she might at any future time repeat the deadly act with impunity. The woman who destroys her new-born infant is definitely and heinously guilty from the first moment that the murderous design is entertained in her thoughts, and she ought to be dealt with on that understanding by all whom it may concern.

In our contention for the recognition of this principle we may seem to be taking a hard view, and one which would involve a result to the unfortunate culprit revolting to public opinion—as a matter of fact there is not the smallest risk that the crime of infanticide will ever in this age be dealt with on the capital charge; a very moderate period of incarceration generally for concealment of birth is all that would follow. Apart from the bad effect on the lower orders, the systematic glossing over of a crime which is the result of unbridled passions, is calculated to have a most pernicious influence generally at a time when the bonds of society are being boldly loosened, even on points that have hitherto been considered absolutely essential to good morals.

